# Human induced pluripotent stem cell-derived cardiomyocytes (iPSC-CMs) for modeling cardiac arrhythmias: strengths, challenges and potential solutions

**DOI:** 10.3389/fphys.2024.1475152

**Published:** 2024-09-12

**Authors:** Jyotsna Joshi, Cora Albers, Nathan Smole, Shuliang Guo, Sakima A. Smith

**Affiliations:** Department of Internal Medicine, Division of Cardiology, College of Medicine, Wexner Medical Center, The Ohio State University, Columbus, OH, United States

**Keywords:** iPSCs, patient-specific iPSC-CMs, *in vitro* arrhythmia models, ion channels, personalized medicine, CRISPR, gene editing, optogenetics

## Abstract

Ion channels and cytoskeletal proteins in the cardiac dyad play a critical role in maintaining excitation-contraction (E-C) coupling and provide cardiac homeostasis. Functional changes in these dyad proteins, whether induced by genetic, epigenetic, metabolic, therapeutic, or environmental factors, can disrupt normal cardiac electrophysiology, leading to abnormal E-C coupling and arrhythmias. Animal models and heterologous cell cultures provide platforms to elucidate the pathogenesis of arrhythmias for basic cardiac research; however, these traditional systems do not truly reflect human cardiac electro-pathophysiology. Notably, patients with the same genetic variants of inherited channelopathies (ICC) often exhibit incomplete penetrance and variable expressivity which underscores the need to establish patient-specific disease models to comprehend the mechanistic pathways of arrhythmias and determine personalized therapies. Patient-specific induced pluripotent stem cell-derived cardiomyocytes (iPSC-CMs) inherit the genetic background of the patient and reflect the electrophysiological characteristics of the native cardiomyocytes. Thus, iPSC-CMs provide an innovative and translational pivotal platform in cardiac disease modeling and therapeutic screening. In this review, we will examine how patient-specific iPSC-CMs historically evolved to model arrhythmia syndromes in a dish, and their utility in understanding the role of specific ion channels and their functional characteristics in causing arrhythmias. We will also examine how CRISPR/Cas9 have enabled the establishment of patient-independent and variant-induced iPSC-CMs-based arrhythmia models. Next, we will examine the limitations of using human iPSC-CMs with respect to *in vitro* arrhythmia modeling that stems from variations in iPSCs or toxicity due to gene editing on iPSC or iPSC-CMs and explore how such hurdles are being addressed. Importantly, we will also discuss how novel 3D iPSC-CM models can better capture *in vitro* characteristics and how all-optical platforms provide non-invasive and high- throughput electrophysiological data that is useful for stratification of emerging arrhythmogenic variants and drug discovery. Finally, we will examine strategies to improve iPSC-CM maturity, including powerful gene editing and optogenetic tools that can introduce/modify specific ion channels in iPSC-CMs and tailor cellular and functional characteristics. We anticipate that an elegant synergy of iPSCs, novel gene editing, 3D- culture models, and all-optical platforms will offer a high-throughput template to faithfully recapitulate *in vitro* arrhythmogenic events necessary for personalized arrhythmia monitoring and drug screening process.

## 1 Introduction

The human cardiac junctional dyad is a highly evolved signal amplification and transduction system with an intricate assembly of ion channels and membrane proteins. In concert, the sarcoplasmic reticulum (SR) and transverse tubule (TT) translates the wave of sarcolemmal depolarization to the TT to induce calcium-induced calcium release (CICR) from internal SR stores leading to excitation-contraction (E-C) coupling (Scoote et al., 2003; Manfra et al., 2017; Lu and Pu, 2020). Ion channels and membrane proteins play fundamental roles in maintaining E-C coupling, and cardiac membrane cytoskeletal proteins and adapter proteins, such as spectrins and ankyrins, play a critical role in the post-translational targeting and localization of key proteins, such as the sodium/calcium exchanger (NCX), sodium/potassium ATPase (Na/K-ATPase), inositol triphosphate receptor (InsP3R), and ryanodine receptor 2 (RyR2) (Smith et al., 2015; El Refaey and Mohler, 2017; Derbala et al., 2018). However, mutations in the genes encoding ion channels and myocyte structural components (e.g., Ankyrins, βII spectrin, Connexin 43, COL12A1 and ANGPTL2), disturbs cardiac electromechanical homeostasis and contribute to cardiac channelopathies and inherited arrhythmias (Giudicessi and Ackerman, 2013; Musa et al., 2016; Lozano-Velasco et al., 2020; Wang and Tu, 2022). An important example is Brugada syndrome (BrS), an inherited channelopathy caused by mutations on the SCN5A gene encoding cardiac sodium channel, which has a prevalence of 1–2,000 to 1 to 5,000 (Chockalingam and Wilde, 2012; Mariani et al., 2024). It is characterized by abnormal ECG recordings (ST-segment elevation) and accounts for ∼4–12% of sudden cardiac deaths cases in children and young athletes and 10%–20% of sudden cardiac deaths in infant (Chockalingam and Wilde, 2012; Mariani et al., 2024). Similarly, catecholaminergic polymorphic ventricular tachycardia (CPVT) is a rare but fatal channelopathy that has a prevalence of ∼1 per 10,000 (Tester et al., 2012; Jiménez-Jáimez et al., 2015; Lahrouchi et al., 2017). It is caused by mutations on RyR2 and calsequestrin 2 (CASQ2) genes that can cause sudden cardiac death, especially in pediatric populations (Mariani et al., 2024). Notably, a highly lethal short QT syndrome (SQTS) has a cumulative risk of cardiac events of 40% by 40 years and has a prevalence of 0.02%–0.1% in adults (Ferrero-Miliani et al., 2010; Guerrier et al., 2015). Thus, the prevalence and lethality associated with these inherited arrhythmias underscore the need to deeply understand their mechanistic pathways and establish novel therapies to treat such fatal conditions.

In addition to the genetic mutations, epigenetic regulations caused by non-coding RNAs, DNA methylation and histone modifications are also being implicated in the pathogenesis of arrhythmias (Lozano-Velasco et al., 2020; Wang and Tu, 2022). Additionally, insults imposed by metabolic risk factors, such as diabetes, impairs cardiac voltage-gated ion channels and put patients at increased risk of developing diabetic cardiomyopathy, ventricular dysfunction, arrhythmias, and heart failure (Ozturk et al., 2021). Also, anti-cancer drugs, such as Tyrosine Kinase Inhibitors, pose a substantial threat of QT prolongation and atrial fibrillation (AFib) for cancer patients (Cheng et al., 2021). Notably, inherited cardiac channelopathies (ICC) often exhibit incomplete penetrance and variable expressivity while gender, racial, ethnic, and social factors also cause variable susceptibility to develop cardiac diseases, clearly indicating the intricate pathogenic signaling web and the need to establish personalized cardiac disease modelling and therapeutic screening (Giudicessi and Ackerman, 2013; Graham, 2015; Ntusi and Sliwa, 2021).

Human cardiac tissue is the gold standard to understand patient-specific disease mechanisms; however, it is not feasible to consistently obtain invasive biopsies of cardiac tissues from patients nor it is an scalable approach to utilize the short-lived extracted cardiomyocytes for multiple mechanistic and therapeutic studies (Nijak et al., 2021). Thus, it is essential to have an accessible and scalable *in vitro* model system that will reflect the phenotypic characteristics of human cardiomyocytes and carry genomic traits of an individual patient. In this respect, heterologous cell culture systems, including HEK293 cells, Xenopus oocytes and Chinese hamster ovary, provide useful models to understand disease mechanisms (Meijer van Putten et al., 2015). However, such cellular systems do not carry the genome of the individual patient and may lack accessory channel subunits, or other native cardiac proteins, present in the human cardiomyocytes (Selga et al., 2018). Thus, such heterologous cell cultures may not definitively represent patient-specific disease phenotypes. Additionally, transgenic animals provide vital *in vitro* information but these models do not fully recapitulate the human genome and cardiac electro-pathophysiology (Moretti et al., 2010; Meijer van Putten et al., 2015). For instance, the mouse heart beat is almost 8 times faster than of humans and mouse cardiac repolarization depends mainly on *I*_to_, *I*_K, slow1_, *I*_K, slow2_ and *I*_SS_ ion currents while the repolarization in human cardiomyocytes is mainly dependent on *I*_Ks_ and *I*_Kr_ (Garg et al., 2018a). To overcome some of these limitations, human induced pluripotent stem cell-derived cardiomyocytes (iPSC-CMs) have evolved as a promising tool in personalized cardiac disease modeling and drug screening (Yoshida and Yamanaka, 2017).

In this review we aim to provide in-depth information on how iPSCs, iPSC-CMs, and patient-specific iPSC-CMs-based arrhythmia models have evolved in the field and how such iPSC-CMs capture *in vitro* electrophysiological characteristics. Also, we will examine recent developments on 3D disease models, high-throughput screening, all-optical platforms, and patient-independent iPSC-CMs-based arrhythmia models using CRISPR/Cas9 to illuminate how such developments have enabled efficient recapitulation of *in vitro* electrophysiological characteristics in a rapid, non-invasive and high-throughput manner. Next, we will discuss concerns on the structural and functional immaturity of human iPSC-CM and review some novel technologies, such as gene-editing and optogenetics that are instrumental in tuning their functional characteristics. Finally, we will briefly review some recent clinical studies and breakthroughs on the human iPSC-CMs for personalized cardiotoxicity screening and regenerative medicine to provide insight on the translational impact of iPSC-CMs in disease modeling, drug screening and therapeutic medicine. Our overall goal is to understand the application and challenges of human iPSC-CMs and examine novel technologies that can strengthen their scope in arrhythmia modeling.

## 2 Patient-specific/human iPSC-CMs and their applications in modeling arrhythmias

**FIGURE 1:**
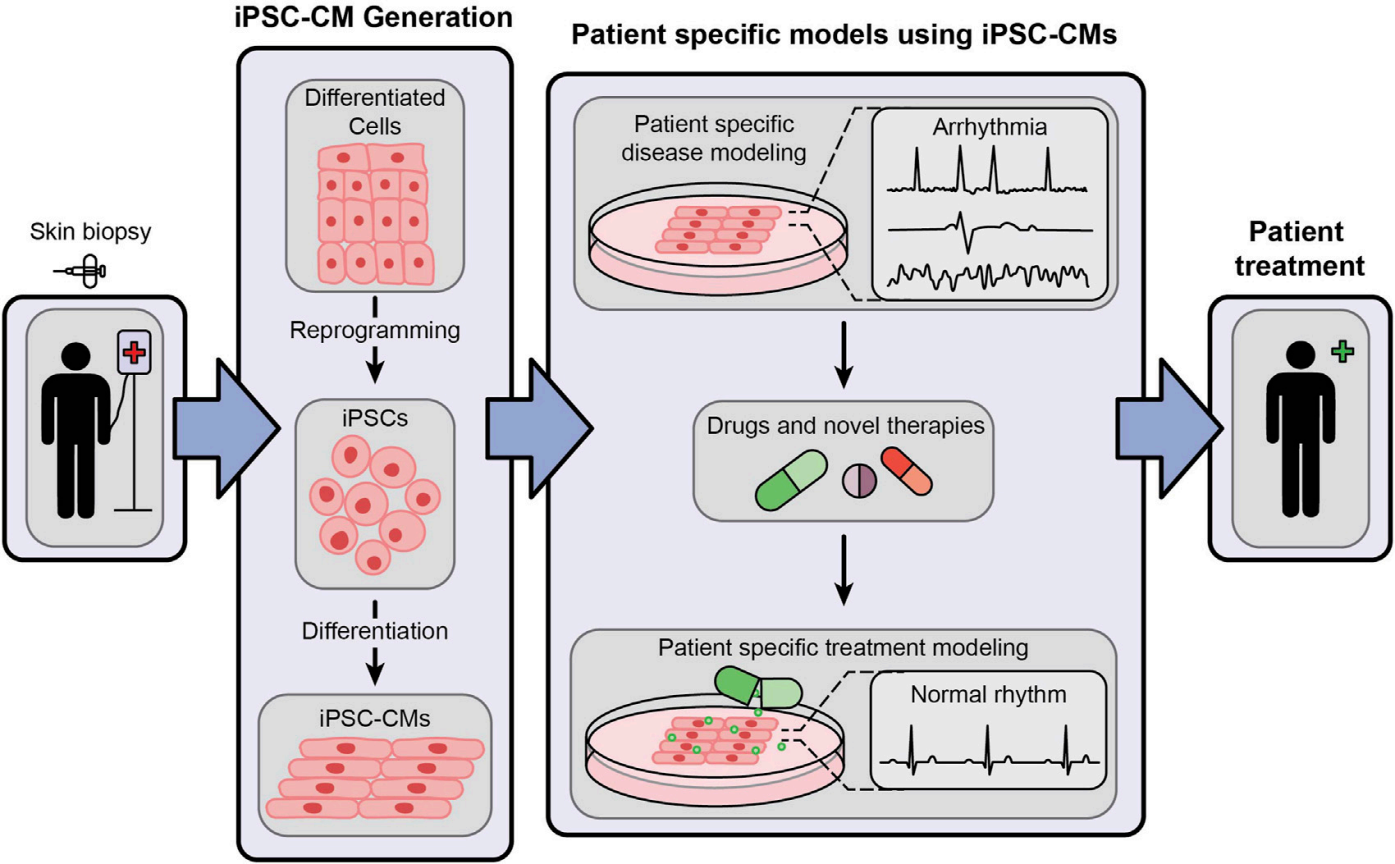
An illustrative representation showing the generation of patient-specific iPSC-CMs and their role in cardiac disease modeling and personalized medicine.

Unlike human embryonic cells, human iPSCs are not hindered by ethical concerns. Interestingly, they are almost inexhaustible and genetically editable, providing an opportunity to create a library of genetic variants causing arrhythmias, establish isogenic controls and identify the pathogenic variants from emerging unknown variants. These methods are crucial for risk stratification in clinical care management, uncover mechanistic signaling pathways, and test potential life-saving therapies to treat specific variant-induced arrhythmogenic syndromes in a high-throughput manner (Hylind et al., 2018; Wu et al., 2019; Huang et al., 2021). The overall role of iPSC-CMs in personalized therapeutic screening, drug development and personalized medicine is illustrated in Figure 1. The iPSC-CMs will greatly aid in the early diagnosis and targeted therapies of ICC and cardiomyopathies, generally threatened by severe ventricular arrhythmias and sudden cardiac arrest (Napolitano et al., 2012; Fernández-Falgueras et al., 2017; Simons et al., 2023). Thus, in the subsequent paragraphs we will review on the early landmark discoveries on iPSCs, patient/human iPSC-CMs, and their evolution on the human arrhythmia modeling.

### 2.1 Early landmark discoveries

**FIGURE 2:**
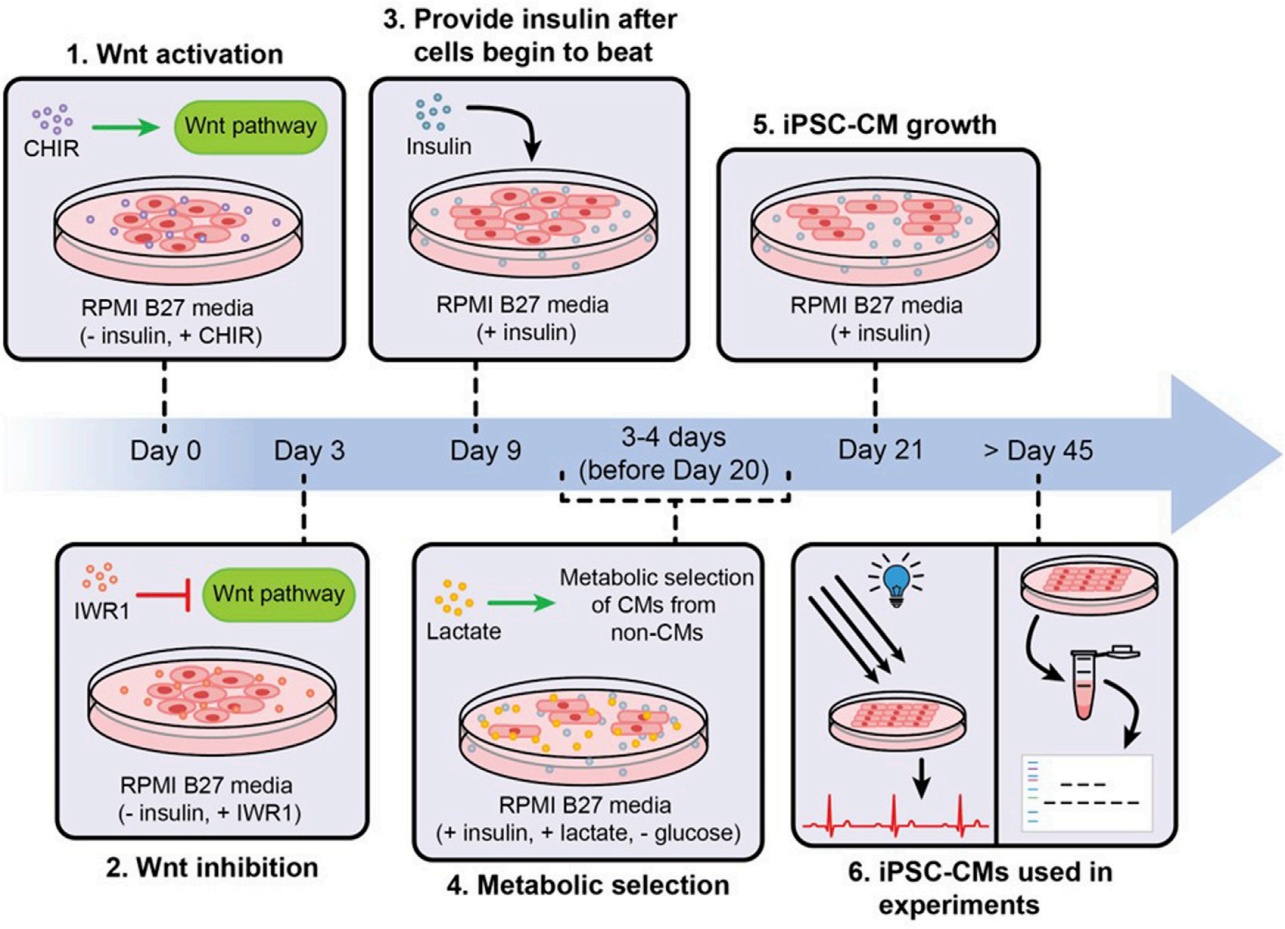
An illustration showing the differentiation, metabolic purification and maturation of iPSC-CMs. iPSCs are differentiated into iPSC-CMs via biphasic activation and inhibition of Wnt signaling, non-iPSC-CMs were removed from cultures via metabolic purification, and the purified iPSC-CMs were allowed to grow and mature via long-term culture.

In early 2006, Takahashi and Yamanaka identified four critical transcription factors, known as Yamanaka factors- Oct3/4, Sox2, Klf4 and c-Myc (OSKM) and they were able to reprogram skin fibroblasts of mice into iPSCs (Takahashi and Yamanaka, 2006). They later found that the OSKM cocktail can also reprogram human fibroblasts into iPSCs (Takahashi et al., 2007). Since then, enormous work has been done to improve iPSC reprogramming, enhance their pluripotency and improve their differentiation (Romito and Cobellis, 2016; Liu et al., 2020). Thus, as of now, iPSCs can be derived from virtually any mature somatic cell of a patient, such as from blood, skin, hair or urine samples, and can be differentiated into cells of all three germ layers, such as neuronal cells, pancreatic cells, alveolar cells, hepatocytes, blood cells, kidney tubule cells, skeletal muscle cells, and cardiomyocytes (Adegunsoye et al., 2023). For instance, a flowchart depicting the differentiation of iPSCs into iPSC-CMs via biphasic activation and inhibition of Wnt signaling (Joshi et al., 2022) is illustrated in Figure 2. Importantly, following reprogramming, iPSCs retain the genomic traits of the donor cells, reflect the phenotypic differences caused by variations in the genetic makeup, and are immunologically similar to the host cells (Pourrier and Fedida, 2020; Micheu and Rosca, 2021; Adegunsoye et al., 2023). This has allowed patient-specific iPSC-CMs to closely mimic the genetic, cellular and immunological characteristics of the disease phenotype.

### 2.2 Early Utilization of iPSC-CMs for Disease Modeling.

Nearly 15 years ago, the Karl-Ludwig Laugwitz lab became the first group to utilize patient-derived iPSC-CMs for modeling a long-QT syndrome (LQTS), an ICC characterized by prolonged QT interval, ventricular tachyarrhythmia and sudden cardiac death (Moretti et al., 2010). In their study, they screened a family affected by LQTS 1 and identified a mutation in the KCNQ1 gene (R190Q) that encodes the delayed rectifier I_Ks_ current. They obtained dermal fibroblasts from two family members and from two healthy controls, reprogramed them into iPSCs, differentiated them into iPSC-CMs, and finally characterized the cells. The action potential duration (APD) of the ventricular and atrial cells, derived from LQTS patients, were significantly prolonged when compared to those derived from healthy controls. In addition, R190Q-KCNQ1 mutation displayed severe channel trafficking defects and the mutated iPSC-CMs displayed 70%–80% reduction in *I*_KS_ current and higher susceptibility to isoproterenol-induced tachyarrhythmia, which was rescued with beta blocker (Moretti et al., 2010). Thus, this was the first study to provide evidence regarding the scope of iPSC-CMs in recapitulating patient-specific channelopathy *in vitro*.

In 2011, the Chris Denning lab examined the scope of iPSC-CM as an *in vitro* humanized drug evaluation platform for inherited LQTS (Matsa et al., 2011). They reprogrammed iPSCs from skin fibroblasts of a patient with LQTS type 2, having a mutation in KCNH2 gene (G1681 A) (that encodes the potassium ion channel *I*_Kr_), differentiated them into functional cardiomyocytes and compared the sensitivity of control iPSC-CM vs. the patient-derived LQT2-iPSC CM with agonists and antagonists of β-adrenergic receptors and of potassium channels. They found that LQT2–iPSC-CM exhibited prolonged APD and increased sensitivity to early after depolarizations (EADs) with isoprenaline treatment, compared to control iPSC-CM, and such events were rescued by β-blockers. In addition, *I*_Kr_ channel blocker (E4031) caused EADs in 30% of the LQT2–iPSC-CM while the potassium channel enhancers (nicorandil and PD-118057) shortened APD by more than 17%, demonstrating the efficacy of these drugs in normalizing the prolonged repolarization of CM with mutation in KCNH2 gene (Matsa et al., 2011). In another study, the Šaric group examined the utility of iPSC-CMs, derived from patients having RYR2 mutation (Fatima et al., 2011). They reported that iPSC-CMs derived from dermal fibroblasts of patient with inherited CPVT1 (caused by mutation in the RYR2 p.F2483I), exhibited arrhythmias and delayed afterdepolarizations (DADs) after catecholaminergic stimulation. Compared to control iPSC-CMs, CPVT1-iPSC-CMs demonstrated higher amplitudes and longer durations of spontaneous Ca^2+^ release events at basal state and the CICR events continued after repolarization, and such phenomena were eliminated with cytosolic cAMP levels with forskolin (Fatima et al., 2011). These foundational studies highlight the ability of patient-derived iPSC-CMs in modeling variant-induced arrhythmogenesis *in vitro*.

### 2.3 The growth of iPSC-CM modeling for multiple variants

Since aforementioned pioneering studies, numerous other studies have shown the ability of patient-derived iPSC-CMs in capturing specific variant-induced arrhythmias, such as LQTS, SQTS, CPVT, Timothy syndrome, and BrS, which are the results of genetic mutations in cardiac ion channels, transporters, or key cardiac proteins, such as KCNQ1 (LQTS1), KCNH2 (LQTS2), RYR2 (CPVT1), CASQ2 (CPVT2) (van Mil et al., 2018; Pourrier and Fedida, 2020). Sun et al., 2023 reported that iPSC-CMs, derived from a SCN5A mutation-positive (D356Y) BrS family with severely affected proband, showed higher arrhythmias, slower depolarization and irregular calcium signaling when compared to controls. Furthermore, iPSC-CMs derived from asymptomatic mutation carriers were milder than that of proband iPSC-CMs, highlighting the ability of patient-specific iPSC-CMs in revealing phenotypic severity of BrS (Sun et al., 2023). Notably, CRISPR has provided an opportunity to rapidly expand the scope of iPSC-CMs in disease modeling and establish patient-independent human iPSC-CM-based models to uncover mechanistic pathways of genetic variants causing arrhythmias. For example, Chavali et al. introduced a CACNA1C-p.N639T variant in an unrelated but already established healthy human iPSC line using CRISPR/Cas9, differentiated them into iPSC-CMs and reported that the variant showed prolonged action potential and slowing of voltage-dependent inactivation of Ca_V_1.2 currents (Chavali et al., 2019). Similarly, Shafaattalab et al. introduced a heterozygous I79N ± TNNT2 variant into human iPSCs, differentiated them into iPSC-CMs and demonstrated that the variant iPSC-CMs exhibited- 1) lower intracellular Ca^2+^ transients, 2) clear pattern of alternans for both voltage (V_m_) and Ca^2+^ transients when paced at frequencies >75 bpm, 3) significant upregulation of atrial natriuretic peptide, brain natriuretic peptide, ECM remodeling and Notch signaling components, when compared to the isogenic controls (Shafaattalab et al., 2021). In another study a homozygous Na_V_1.5 KO iPSC line, established using CRISPR/Cas9 and differentiated into cardiomyocytes showed: 1) lower Na^+^ currents, 2) reduced action potential amplitudes, and 3) delayed propagation of Ca^2+^ waves, where such abnormal electrophysiological functions were rescued by WT Na_V_1.5 channel expression in the KO iPSC-CMs (Pierre et al., 2021). Additional findings from foundational studies and current studies on human/patient-specific iPSC-CMs to understand the role of ion channel mutations and their functional modifications during channelopathies are provided in Supplementary Table 1. In brief, iPSC-CMs recapitulate the *in vitro* electrophysiological characteristics of ionic currents, action potentials, calcium transients, proarrhythmogenic and arrhythmogenic events, and provide physiologically relevant disease models and drug screening platforms (van Mil et al., 2018; Pourrier and Fedida, 2020; Micheu and Rosca, 2021).

### 2.4 Pros and cons of human iPSC-CMs versus other models

Human/patient-specific iPSC-CMs carry human/patient-specific genome, reflect human cardiac electrophysiology, and provide an almost unlimited supply of cells for investigation. Thus, iPSC-CMs are preferred in disease modeling over heterologous cell cultures and animal models (Table 1). For example, Selga et al. (2018) compared the functional characteristics of sodium current in iPSC-CMs derived from a patient diagnosed with BrS and having a mutation in the SCN5A gene (c.1100G>A), with those from transfected tsA201 cells, carrying the same mutation. They reported that patient-derived iPSC-CMs showed: 1) a significant reduction (45%) in the peak *I*_Na_ density, 2) the I_Na_-voltage activation curve was shifted by 7.42 mV and 3) the *I*_Na_-voltage inactivation curve was negatively shifted by 8.5 mV compared to the control iPSC-CMs. Interestingly, transfected tsA201 cells also showed a significant drop in peak *I*_Na_ by ∼ 50% when compared to wild type (WT) controls, but these cells did not show any voltage-dependent changes in kinetic properties of the sodium current that was observed in patient and control iPSC-CMs. Thus, the patient-specific genetic background, not just the specific gene variant, could affect overall expression of cardiac-specific proteins and thereby guide bio-functional characteristics of the ion channel currents (Selga et al., 2018).

**TABLE 1:** A comparison on the advantages and disadvantages of animal models, heterologous cell culture models and iPSC-CMs in arrhythmias research.

	Advantages	Disadvantages
Animal Models	• Abundance of well-established and well-studied disease models • Established and novel transgenic technology, making it easier to create genetically altered models • Small animal models, including mice and rats, are relatively inexpensive and widely used, making protocols and techniques easily accessible (Kawaguchi and Nakanishi, 2023)	• Large animal models have similarities to human heart structure and electrophysiology but raise ethical concerns (Burnett et al., 2021) • Rodent models display significant differences in cardiac physiology as compared to humans (Davis et al., 2011; Joukar, 2021) • Animal models do not completely reproduce human disease phenotypes (Davis et al., 2011)
Heterologous Cell Culture	• Allow for evaluation of functional changes when a gene is mutated or inserted, as there is always a control line available • Immortalized cell lines are well-documented and easy to obtain (Lippi et al., 2020)	• Proteins that are inserted into another cell lack the cellular context of a human cardiomyocyte (Davis et al., 2011)
iPSC-CMs	• Similar electrophysiology to that of normal human cardiomyocytes (Burnett et al., 2021) • CRISPR editing allows for generation of isogenic controls (Han and Entcheva, 2023) • Genetically identical to the individual they were derived from- allows for personalized medicine approaches to disease modeling (van den Brink et al., 2020) • Unlimited quantity, due to self-renewing capability of iPSCs	• Display immature phenotypes in terms of their morphology, cytoskeleton, and ion channel characteristics (Barbuti et al., 2016)

Nevertheless iPSC-CMs are hindered by their immature structural and ion channel characteristics when compared to native cardiomyocytes. To address such limitations, recent developments in cell reprogramming, differentiation and maturation strategies (Section 5) have positively impacted their scope in clinical studies for disease modeling and drug screening. As an example, a recent clinical study “Modeling and Pharmacological Targeting of Genetic Cardiomyopathy in Children Via Cardiomyocytes Derived From Induced Pluripotent Stem Cells” (study location- France) was designed to generate patient-specific iPSCs and iPSC-CMs to study the molecular mechanisms of cardiomyopathies generated due to genetic defects [NCT03696628]. In the study, iPSCs were generated from peripheral blood mononuclear cells (PBMCs), using integration-free Sendai virus gene-delivery method, from three different Duchenne Muscular Dystrophy patients (<17 years, harboring deletions of exons 1, 52 and 55 in the dystrophin gene). The reprogrammed iPSCs showed normal karyotypes, pluripotency status and differentiated into iPSC-CMs using embryoid body formation (Souidi et al., 2020). Thus, although there is room for improvement, human/patient-specific iPSC-CMs provide an unlimited and instrumental platform in modeling arrhythmias due to multiple emerging variants and in understanding their mechanistic pathways.

## 3 Roles of 3D culture, gene editing and all-optical platforms in arrhythmia research

### 3.1 Tissue engineering platforms and beyond

The emergence of 3D models of iPSC-CMs, such as organoids, cell sheets and engineered heart tissues (EHT), have provided iPSC-CMs with additional methodologies to mimic *in vitro* cardiac events, such as tissue contractility, AP conduction velocity, origin, and propagation of re-entrant waves that are not attainable in traditional 2D cultures (Simons et al., 2023). Notably, the Lior Gepstein lab reported that iPSC-CMs, derived from dermal fibroblasts from a symptomatic SQTS patient having an N588K variant in the KCNH2 gene exhibited: 1) shortened APD, 2) increased *I*_Kr_ current density, and 3) increased channel protein expression compared to the healthy and isogenic controls at the cellular level (Shinnawi et al., 2019). Using optical mapping techniques, it was found that iPSC-CM-derived cardiac cell sheets exhibited shortened APD and increased susceptibility for sustained spiral waves at the tissue level, and such arrhythmogenic events were rescued with quinidine and disopyramide but not with sotalol. In another study, Goldfracht et al. examined the utility of EHT for modeling LQTS2 and CPVT2 (Goldfracht et al., 2019). They created EHTs by combining healthy or patient-specific iPSC-CMs with an ECM gel (derived from porcine cardiac tissues), allowed the cell-gel mixture to condense as rings within circular casting molds and they were transferred them into a passive stretching device for maturation. The optical recording data demonstrated significantly longer APD values measured in the LQTS2 derived EHT compared to their healthy control counterparts. Also, the CPVT2-ECM derived EHTs, but not their healthy counterparts, showed irregular calcium transients upon isoproterenol treatment (10 µm) and the occurrence and severity of such abnormal calcium activity was significantly lower when compared to the single-celled CPVT2-iPSC-CM counterparts, possibly due to higher electrotonic interactions in 3D. This implies 3D models are more clinically relevant in capturing *in vitro* electrophysiological features, when compared to 2D, as very frequent and very severe arrhythmogenic responses seen with single cells are often incompatible with life (Goldfracht et al., 2019).

### 3.2 Identification and validation of new variants with genetic testing

The incorporation of genetic testing as a clinical component for ICC patients has enabled the identification of hundreds of novel variants. However, this poses a significant challenge of identifying numerous emerging variants of uncertain significance (VUS) as normal or pathological, which is crucial for early risk stratification in clinical care (Garg et al., 2018b; Hylind et al., 2018). It has been reported that iPSC-CMs derived from a LQT2 patient having a novel missense variant (T983I) in the KCNH2 gene exhibited: 1) a significant prolongation in APD, 2) reduction of *I*_Kr_, 3) reduction of hERG protein levels, and 4) higher pro-arrhythmogenic events compared to its healthy counterparts (Garg et al., 2018b). Notably, precise genomic correction of VUS iPSC-CM using CRISPR/Cas9 rescued the abnormal electrophysiological behavior while the introduction of the homozygous variant in the healthy cells elicited hallmarks of LQTS, implicating the pathogenic role of the KCNH2^T983I^ variant (Garg et al., 2018b).

Thus, CRISPR-based genome editing tools can greatly aid in the early identification of unknown arrhythmogenic variants. Moreover, novel CRISPR-based interference and activation system (CRISPRi/a) have emerged to overcome the concerns of double strand break-induced cellular toxicity and permanent gene-editing-induced phenotypic changes in iPSCs and iPSC-CMs (Haapaniemi et al., 2018; Tian et al., 2019). CRISPRi/a uses deactivated Cas9 (dCas9) enzyme linked to an effecter (an activator or a repressor) of gene transcription along with guide RNA (gRNA) provides reliable, reversible and spatiotemporal control of genetic modulation. In this regard, a recent study showed no significant effect of the CRISPRi components (DOX and dCas9-KRAB), on protein and mRNA levels of the ion channels of interest nor on the specific electrophysiological parameters in iPSC-CMs and clearly delineates that the observed physiological effects (such as APD80 prolongation and slowing of conduction velocity) were solely due to CRISPRi knockdown of KCNJ2 and GJA1 respectively in iPSC-CMs when gene edited using the DOX-inducible (Tet-on) dCas9-KRAB system (Han and Entcheva, 2023). Thus, novel CRISPR-based methods demonstrate a reliable gene editing platform to faithfully decipher gene-phenotype relations for arrhythmia research.

### 3.3 Novel use of all-optical platforms for iPSC-CM modeling

There is growing concern regarding the increasing cost, lengthy time, and the explosion in the number of candidate drugs to be tested for emerging arrhythmogenic variants. While classic patch-clamp electrophysiology requires physical contact and is a low throughput method, current automated systems significantly enhance the throughput, such as Multichannel Electrode Arrays (MEA), xCELLigence, and Fluorometric Imaging Plate Reader (FLIPR). Yest these systems cannot characterize tissue-level or multicellular effects, in 2D or 3D, respectively (Klimas et al., 2016). Fortunately, the birth of all-optical platforms, which is a merger of optogenetics and optical mapping methods, provides an opportunity for contactless actuation and readout of electrophysiological signals, offering tremendous potential for high-throughput functional readout for variant screenings and drug discovery (Klimas et al., 2016; Baines et al., 2024). Notably, the Emilia Entcheva lab has previously described an automated all-optical system (OptoDyCE) for capturing cardiac electrophysiology at the cellular/multicellular level (Klimas et al., 2016). This innovative system utilizes: 1) low-power LED light sources, 2) iPSC-CMs expressing light-gated cation channel Channelrhodopsin (iPSC-CMs-ChR2) where ChR2 acts as optogenetic actuator to enable optical pacing of the human iPSC-CMs, 3) optical sensors to record voltage (di-4-ANBDQBS) and intracellular calcium (Rhod-4a.m.) signals from the human iPSC-CMs, 4) multicellular samples in 96-wells, in 2D or 3D format, for HT capabilities, 5) a custom-built inverted microscope, high speed camera, and 6) custom-software for data analysis. As proof of concept, the system was able to perform dynamic drug-dose testing (using multi-beat pacing protocol) on 96-well format in less than 10 min, with the ability to record >30,000 cell readouts per 96-well plate, examine records at both global and cellular scale and foun pro-arrhythmic risks from voltage, calcium or contraction responses (Klimas et al., 2016). Recently the Entcheva lab has demonstrated another low-cost OptoDyCE-plate that can be used for the readout of cardiac function, providing data on change in APD and calcium transient duration (CTD) in human iPSC-CMs under spontaneous and paced, electrical or optogenetic (utilizing iPSC-CMs-ChR2 cells), conditions in standard 96 (or 3D electrospun PCL isotropic and anisotropic wells) and 384-well plate formats (Heinson et al., 2023). Using this system, investigators successfully tested the action of 4 drugs, dofetilide, cisapride, vanoxerine and nifedipine, on human iPSC-CMs and reported that:- 1) the first 3 drugs induced pacing frequency-dependent APD80 prolongation while the latter drug shortened APD80, 2) all 4 drugs showed pronounced APD effect at slower pacing rates while the effects were reduced at higher pacing rates, and 3) CTD80 changes under paced conditions were much smaller with the drugs (Heinson et al., 2023). These reports clearly depict that a synergy of iPSC-CMs, gene editing, high-throughput platform, and all-optical system would allow acquisition of massive amount of electrophysiological data, under static or dynamic conditions, in a non-invasive and real-time manner; thus offering a crucial platform to rapidly and efficiently capture electrophysiological features of multiple variants while simultaneously screening candidate drugs. Additional findings on such state-of-art iPSC-CM-based arrhythmia studies utilizing 3D models, CRISPR-Cas9 mediated gene editing, optogenetics, and optical platform are summarized in Table 2. In summary, by utilizing iPSC-CMs, 3D-culture models, CRISPR-based gene modulation, and all-optical platform, it is feasible to obtain real-time and high-throughput physiologically relevant electrophysiological data that offers unique potential in gaining mechanistic insights for emerging arrhythmia variants *in vitro* and will provide innovative platform for the discovery of novel therapies.

**TABLE 2:** State-of-art iPSC-CM-based arrhythmia studies utilizing 3D models, CRISPR-Cas9 mediated gene editing, Optogenetics and Optical mapping platform.

BrS	Liang et al. (2016)	SCN5A p. (Arg620His and Arg811His) SCN5A 1397Δ	1. Abnormal AP profiles 2. Reduction of I_*Na*_ amplitude 3. Increased variation of beating intervals 4. Abnormal Ca^2+^ transients 5. CRISPR-Cas9 mediated gene-correction of BrS mutation caused recovery of some electrophysiological traits
	Sun et al. (2023)	SCN5A p. (Asp356Tyr)	1. Beating interval variation 2. Irregular Ca^2+^ signaling and Ca^2+^ transients 3. CRISPR-Cas9 mediated gene-correction of the Asp356Tyr mutation rescues the arrhythmic phenotype, Nav1.5 expression levels, and sodium channel function in iPSC-CMs
CPVT	Park et al. (2019)	RYR2 p. (Arg4651Ile)	1. CPVT and genome-edited isogenic CPVT iPSC-CMs displayed Ca^2+^ sparks more often than in WT. 2. Utilized optogenetically based-muscular thin films (Opto-MTF) of CPVT. 3. Opto-MTF were stimulated with light pulses (for ChR2 activation) and Ca^2+^ sparks, activation, direction and velocity were optically mapped, with and without isoproterenol 4. Under 3-Hz pacing and isoproterenol, WT opto-MTS showed homogenous Ca^2+^ wave propagation (speed and direction) while CPVT tissued showed circular pattern of Ca wave propagation 5. Heterogenous AP repolarization and excitability in CPVT tissue models
LQTS	Takaki et al. (2019)	KCNQ1 p. (Ala341Val)	1. Established optical recordings of electrophysiology 2. Increased frequency of EADs 3. Prolonged APD
	Maurissen et al. (2024)	KCNH2 p. (Asn588Asp)	1. Reduction of I_*Kr*_, increasing the likelihood of tachyarrhythmia 2. Prolonged Field potential duration (FPD) in both 2D and 3D cardiac tissue sheets
SQTS	Shinnawi et al. (2019)	KCNH2 p. (Asn588Lys)	1. Optical recording of SQTS-hiPSC-derived cardiac cell sheets (CSC) showed shortened APD and increased susceptibility to spiral waves 2. Patch clamping at cellular level showed increased I_*Kr*_ 3. Increased hERG channel protein expression
	Shiti et al. (2023)	KCNH2 p. (Asn588Lys)	1. hiPSC-based atrial tissue model showed shortened APD 2. Increased I_*Kr*_ current density 3. Arrhythmia induction via electrical stimulation yields shorter arrhythmia cycle length as compared to control
	Maurissen et al. (2024)	KCNH2 p. (Asn588Lys)	1. Increased I_*Kr*_ 2. Both 2D and 3D cardiac tissue sheets display reduced FPD

## 4 Challenges of using iPSC-CMs in arrhythmia research and clinical care

### 4.1 Variations in iPSCs

Clearly, human iPSC-CMs are invaluable tools for modeling arrhythmias and in understanding their mechanistic pathways; however, they exhibit some limitations in personalized screening and cardiotoxicity studies. One of the limitations of iPSC-CMs in personalized arrhythmia modeling and mechanistic studies stems from the variations in iPSCs themselves that may arise due to differences in: 1) tissue source and donor characteristics, 2) reprogramming methods, 3) culture conditions, and 4) clonal variations in iPSCs derived from the same donor and generated under the same reprogramming event, all which affects their pluripotency, differentiation potential and phenotypic characteristics (Han and Entcheva, 2023; Nath et al., 2023).

### 4.2 Biophysical characteristics of ion channels

In a recent study, Jonathan M Cordeiro and his group demonstrated that human iPSC-CMs and adult cardiomyocytes show a similar block of sodium current (*I*_Na_) with lidocaine under voltage clamp but not under current clamp, suggesting a clear difference in the biophysical properties of *I*_Na_ (Goodrow et al., 2018). This may be due to the co-presence of fetal (exon 6 A) and adult (exon 6) isoforms of Na_v_1.5 in iPSC-CMs or differences in the ratios of channel pore-forming α and accessory β subunits (the latter facilitates in the trafficking of Nav1.5 to the membrane) in the iPSC-CMs, compared to native cardiomyocytes (Goodrow et al., 2018). Another study reported no difference in *I*_Na_ and action potential upstroke velocity (V_max_) in human iPSC-CM derived from 3 BrS patients who tested negative for coding region mutations in the known BrS-associated genes, when compared to iPSC-CM derived from healthy controls (Veerman et al., 2016). However, in another study (but under the same experimental system), loss of sodium channel function was detected, as reduced *I*_Na_ and *V*_max_ *,* in iPSC-CMs harboring a single sodium channel mutation SCN5A-1795insD (Bezzina et al., 1999). Such findings suggest: 1) ion channel dysfunction, mainly cardiac sodium channel, may not be the sole cause of arrhythmias in BrS (Veerman et al., 2016), 2) there may be possible involvement of other membrane proteins, cytoskeletal proteins or extracellular structural changes, such as fibrosis, in channelopathies, (Paci et al., 2020), and 3) the immature ion channel expression in iPSC-CM may fail to detect subtle differences in ionic currents and their biophysical characteristics in certain arrhythmic syndromes, inferring the need of more detailed analysis of iPSC-CM disease phenotypes, including proteomics and transcriptomics, to model arrhythmias *in vitro* (Simons et al., 2023). Hence, it is critical to conduct the study design and interpret the results on iPSC-CMs in arrhythmia research with care.

### 4.3 Structural, electrophysiological and metabolic immaturity

Another limitation of iPSC-CM in cardiac research stems from the challenge that iPSC-CMs are structurally, electro-physiologically, and metabolically more immature than native cardiomyocytes. iPSC-CMs: 1) lack definitive t-tubules, 2) lack or have rudimentary Ca^2+^ handling components such as RyR2, SERCA, calsequestrin, 3) exhibit slower upstroke velocity, shorter plateau phase or repolarization due to lower levels of Na^+^ and L-type Ca^2+^ channels, 4) possess automaticity due to lower current density of Kir_2.1_-encoding *I*_K1_, conferring them more proarrhythmic and depolarized at resting membrane potential than native cardiomyocytes (Paci et al., 2020; Zhou et al., 2023). A thorough comparison on the ultrastructural, contractile, electrophysiological, and metabolic characteristics of iPSC-CMs with those of the adult cardiomyocytes is presented in Supplementary Table 2. In brief, human iPSC-CMs are limited by their structural and functional immaturity, such as lack of definitive t-tubules, poor Ca^2+^ handling machinery, unrestricted presence of gap junctions all over the cell membrane, depolarized resting membrane potential conferring differences in the activation of voltage-dependent ion channels, differences in the ion channel expression levels etc., which needs careful attention to boost the translational impact of iPSC-CM in disease modeling and regenerative medicine. Thus, it is crucial to implement strategies to address the limitations of iPSCs and post-differentiated iPSC-CMs for reliable arrhythmia research.

## 5 Overcoming the challenges of iPSCs and iPSC-CMs in arrhythmia modeling

**FIGURE 3:**
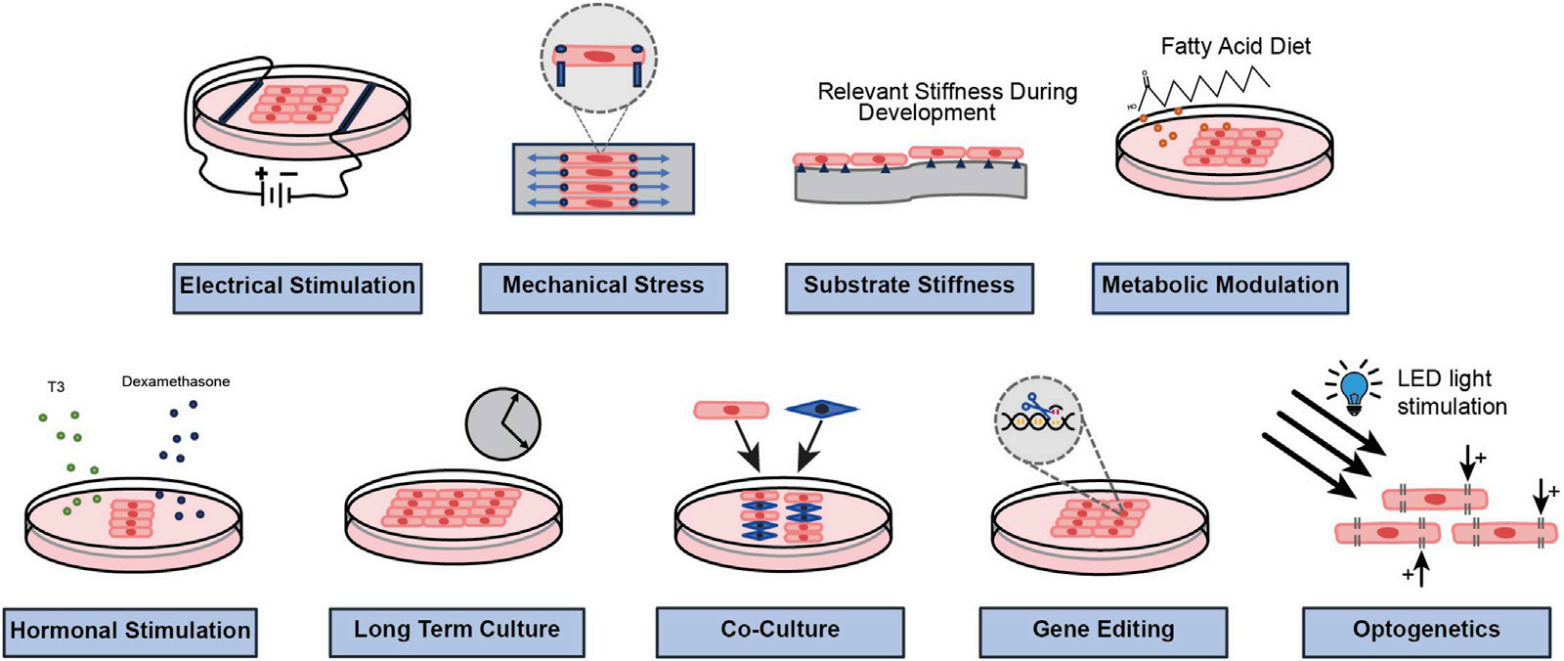
A schematic representation to show common and novel approaches that are being investigated to modify the functional and structural properties of iPSC-CMs.

iPSCs derived from different donors may show phenotypic variations due to multiple genetic and epigenetic differences; hence, it is necessary to have isogenic controls for specific variants to correctly interpret the mechanistic findings (Shinnawi et al., 2019). Additionally, it is crucial to compare results of mutated/diseased iPSC-CMs with their corresponding isogenic controls, produced under same reprogramming and culture conditions (Yoshida and Yamanaka, 2017). Importantly, multiple strategies are being investigated to improve the maturity of iPSC-CMs and reduce their barriers in disease modeling, drug screening and regenerative medicine. Methods, such as long-term culture, electrical stimulation, mechanical stress, substrate stiffness, and biochemical stimulation (e.g., hormonal stimulation, paracrine signaling, metabolic modulation etc.) are being widely investigated to enhance structural and functional maturity in iPSC-CMs (Tu et al., 2018; Wu et al., 2021). We have illustrated some common and novel approaches for functional and structural modification of iPSC-CMs in Figure 3. We will briefly summarize some of the salient points and explore their scope in arrhythmia research and medicine in the following paragraphs.

### 5.1 Culture duration

It has been hypothesized that prolonged cell culture times could promote maturation of iPSC-CMs originated from the fact that it takes almost a decade for native cardiomyocytes *in vitro* to acquire their adult structural phenotypes (Peters et al., 1994; Vreeker et al., 2014). In this aspect, Lundy et al. reported that iPSC-CMs with prolonged culture duration (80–120 days) exhibit more mature structural and functional properties, such as increased cell size and anisotropy, a higher proportion of multinucleated cells, tight and orderly arrangement of myofibrils, a significant improvement in sarcomere contractile performance, and increase in calcium release and reuptake (Lundy et al., 2013). Similarly, other groups have reported the appearance of mature Z-bands, A-bands, H-bands, I-bands when culture duration of iPSC-CMs was increased to 180 days while M-band emerged lately when the culture was further prolonged to a year (Kamakura et al., 2013). Interestingly, it has recently been reported that late-stage iPSC-CM (aged >50 days) post-differentiation shows significantly larger *I*_Ca,L_ density, *I*_Ca,L_ evoked Ca^2+^ transients, and *I*_Na_ and *I*_K1_, conferring them increased upstroke velocity and reduced APD (Seibertz et al., 2023).

### 5.2 Electrical stimulation

Since electrical signals play a fundamental role in the growth and development of the heart, electrical stimulation holds a potential to influence the maturity of iPSC-CM (Wu et al., 2021). Tan et al. reported that an endogenous electrical microenvironment provides structural and functional improvements to iPSC-CM, such as increased expression of cardiac troponin I and synchronized beating of the cardiomyocytes, which they achieved by incorporating trace amount of electrically conductive silicon nanowires (∼0.004% w/v) in otherwise scaffold-free 3D iPSC-CM spheroids (Tan et al., 2015). They further reported that such nano-wired iPSC-CMs synergize with exogenous electrical stimulation as demonstrated by higher 1) cell-cell junction (Cx43, N-cadherin), 2) contractile machinery, 3) Ca^2+^ handling protein RyR2 expression, 4) Ca^2+^ release, but a significant reduction in the spontaneous beating of iPSC cardiac spheroids, addressing a major issue of automaticity with the iPSC-CMs (Richards et al., 2016). Further, Ma et al. reported the scope of cardiac-mimetic exogenous electrical pulses in enhancing the differentiation and functional maturation of iPSC-CMs, which was confirmed by a notable increase in intracellular Ca^2+^ levels and RYR2 expressions via Ca^2+/^PKC/ERK signaling pathway (Ma et al., 2018). Similarly, Yoshida et al. demonstrated that a week regimen of electrical stimulation (2 Hz), using electrically conductive PVA-based permeable microchamber and carbon-based cyto-compatible electrodes, promotes expressions of myocardial structural proteins, such as cardiac troponin T and sarcomere-like structures in iPSC-CMs (Yoshida et al., 2019).

### 5.3 Mechanical stimulation

Given the heart’s intrinsic role as a pump, mechanical stress also holds the potential to significantly influence CM maturity. Leonard et al. reported that progressive incremental mechanical stimulation in the form of applied afterload increases the sarcomere length, cardiomyocyte area and elongation, and calcium handling of iPSC-CMs in EHT, where such benefits plateaus at moderate afterload conditions (0.45 μN/μm) and is pathological at high afterload condition (9.2 μN/μm) (Leonard et al., 2018). Similarly, Abilez et al. (2018) demonstrated that passive stretch, guided by computational modeling, modulates the alignment and calcium dynamics of iPSC-CMs within an engineered heart muscle (EHM). Interestingly, they reported that passive stretch, along with fibroblast co-culture, in 3D EHM and culture duration enhanced the maturity of iPSC-CMs, as elicited by increased gene expression of troponin-T (*TNNT2*), calveolin-3 (*CAV3*), β-adrenergic receptors (*ADRB1*), and potassium (*KCNJ2*) and calcium (*CACNA1C*) ion channels (Abilez et al., 2018).

### 5.4 Biochemical signaling and cellular interactions

Postnatal hormones are known to significantly influence cardiomyocyte maturity (Scuderi and Butcher, 2017). Specifically, the incorporation of thyroid hormones and glucocorticoids have been pivotal in heart maturation during development (Li et al., 2014) (Rog-Zielinska et al., 2015). Triiodothyronine (T3)-treated iPSC-CMs developed elongated morphology, longer sarcomeres, increased mitochondrial activity, and improved calcium handling (Ahmed et al., 2020). Notably, Parikh et al. reported that enriching the iPSC-CM medium with Triiodothyronine (T3) and Dexamethasone (Dex) during days 16–30, followed by single-cell culture on Matrigel mattress for next 5 days, provided 1) development of extensive T-tubule network, 2) promoted RyR2 structural organization, 3) enhanced and uniform calcium release characteristic of E-C coupling in ventricle, and 4) enhanced functional coupling between L-type Ca channels and RyR2 (Parikh et al., 2017). Apart from biochemical signaling, direct cellular interactions or indirect paracrine signaling in a native cardiac tissue are also known to influence cardiomyocyte maturity (Talman and Kivelä, 2018). Thus, iPSC-CM maturation strategies have also utilized methods to mimic those cellular interactions *in vitro*, such as by co-culturing iPSC-CM with endothelial cells, cardiac fibroblasts, epicardial cells, and smooth muscle cells (Yang et al., 2023). Interestingly, Kowalski et al. recently reported that co-culturing immature human iPSC-CMs with mouse sympathetic ganglion neurons (SN) promotes gene expression of mature cardiac structures (e.g., TNNT2, MYL2), ion channels (KCNH2, KCNQ1, KCNJ2, SCN5A), Na^+^-Ca^2+^ exchanger (SLC8A1), calcium handling machinery (ATP2A2, RYR2, PLN) whereas culture of cardiomyocytes with isoproterenol alone did not cause changes in gene expression, inferring the vital role of direct cell-cell interactions in cardiomyocyte maturation (Kowalski et al., 2022). Further, they reported that such iPSC-CM, co-cultured with SN, elicited higher connexin-43 gap junction expression, sarcomere organization, and higher amplitudes of calcium transients but slower calcium relaxation compared to monoculture controls (Kowalski et al., 2022).

### 5.5 Gene editing

Gene editing provides an opportunity to directly probe the genomic characteristics and thereby influence the expression and function of the ion channels, dyad membrane proteins or other structural proteins, and tailor electrophysiological, structural and metabolic changes in iPSC-CMs. For example, Zhou et al. (2023) reported that iPSC-CMs, when overexpressed with KCNJ2 via lentiviral transduction method, showed significant protein expression of K_ir_2.1 and current density of K_ir_2.1-encoding I_K1_. They also showed that such K_ir_2.1-over expressed iPSC-CMs acquired quiescent phenotypes, which required pacing to elicit action potentials and the induced action potentials were abrogated by I_K1_ blocker BaCl_2_, clearly showing the precise functional effect of the K_ir_2.1 gene overexpression on iPSC-CMs (Zhou et al., 2023). They also reported that KCNJ2 overexpression significantly hyperpolarized the diastolic potential, shortened APD, increased maximal upstroke velocity, improved Ca^2+^ signaling, and mitochondrial energy metabolism of the iPSC-CMs (Zhou et al., 2023). In this aspect, Meijer van Putten et al., 2015, reported the role of I_*K1*_ in improving maturity of iPSC-CMs by injecting *in silico* I_k1_, with a peak outward density of 4–6 pA/pF, to the spontaneously beating iPSC-CMs and showed that the cells exhibited 1) ventricle-like action potential morphology, 2) improved resting membrane potential towards the physiological range (−80 mV), 3) increased maximum upstroke velocity (>150 V/s), and 4) proarrhythmic action potential changes, upon injection of both loss-of-function and gain-of-function I_K1_, associated with short QT syndrome and Andersen-Tawil syndrome (Meijer van Putten et al., 2015). Interestingly, Vaidyanathan et al. demonstrated that iPSC-CMs, with adenoviral-mediated I_K1_-enhancement, showed 1) absence of spontaneous beating, 2) stable resting membrane potentials at around −80 mV, and 3) twofold increase in cell size and membrane capacitance. Notably, such I_K1_-enhanced iPSC-CMs, harboring F97C-CAV3 long QT9 mutation, showed increase in APD and late sodium current, representing a more mature arrhythmia model with I_K1_ enhancement (Vaidyanathan et al., 2016). In another study, using transcription activator-like effector nuclease (TALENs)-based gene editing platform, Wheelwright et al. induces expression of adult cardiac troponin I isoform in iPSC-CM and showed that iPSC-CMs depicted adult-like cardiac contractile features, such as faster relaxation kinetics under baseline and β-adrenergic stimulation, when compared to unedited iPSC-CMs (Wheelwright et al., 2020). These studies suggest that gene editing can tailor the expression and characteristics of ion channels and of other cardiac proteins to confer maturity to iPSC-CM for reliable arrhythmia modeling and regenerative medicine.

### 5.6 Optogenetics

An early study by Professor Yue and his team in 2014 demonstrated the ability to optically manipulate and map action potentials of neonatal rat ventricular cardiomyocytes (NRVM) by using excitatory/ inhibitory optogenetic actuators and all-optical system (Park et al., 2014). In brief, using blue light pulses (447 nm, 7.2 mW/mm^2^, 51 ms), they elicited action potentials on electrically unstimulated NRVM, expressing light-gated cation channel (ChR2), and simultaneously mapped their voltage signals via optical activation (655 nm) of red-shifted voltage dye (PGH1). They also demonstrated that electrically stimulated action potentials in NRVM, expressing eNpHR3.0 (an improved version of light-driven chloride pump halorhodopsin), were silenced with green light pulses (1.4–0.67 mW/mm^2^, 530 nm) (Park et al., 2014). Interestingly, they also reported that electrically paced ChR2-expressing cardiomyocytes showed APD prolongation when illuminated with blue light (447 nm) during the repolarization phase (Park et al., 2014). Furthermore, Professor Spundich and his team reported that NRVM cardiomyocytes expressing anion channelrhodopsin from Guillardia theta (GtACRs), showed shortening in APDs when illuminated with green light pulses (510 nm) during the repolarization phase, where the extent of APD shortening was directly proportional to the light intensity (0–230 µW/ mm^2^) (Govorunova et al., 2016). Although these pioneering studies were done on NRVM, these studies clearly depict: 1) how electrophysiological characteristics of iPSC-CMs can be probed via light-activated opsins, which induces light-dependent reversible, unipolar depolarizing (via ChR2 activation) or hyperpolarizing (via GtACRs activation) currents, 2) the implications of such optogenetic tools in basic cardiac research and disease modeling (such as LQTS and SQTS), and 3) the scope of all-optical system in capturing real-time and non-invasive cellular electrophysiology.

**FIGURE 4:**
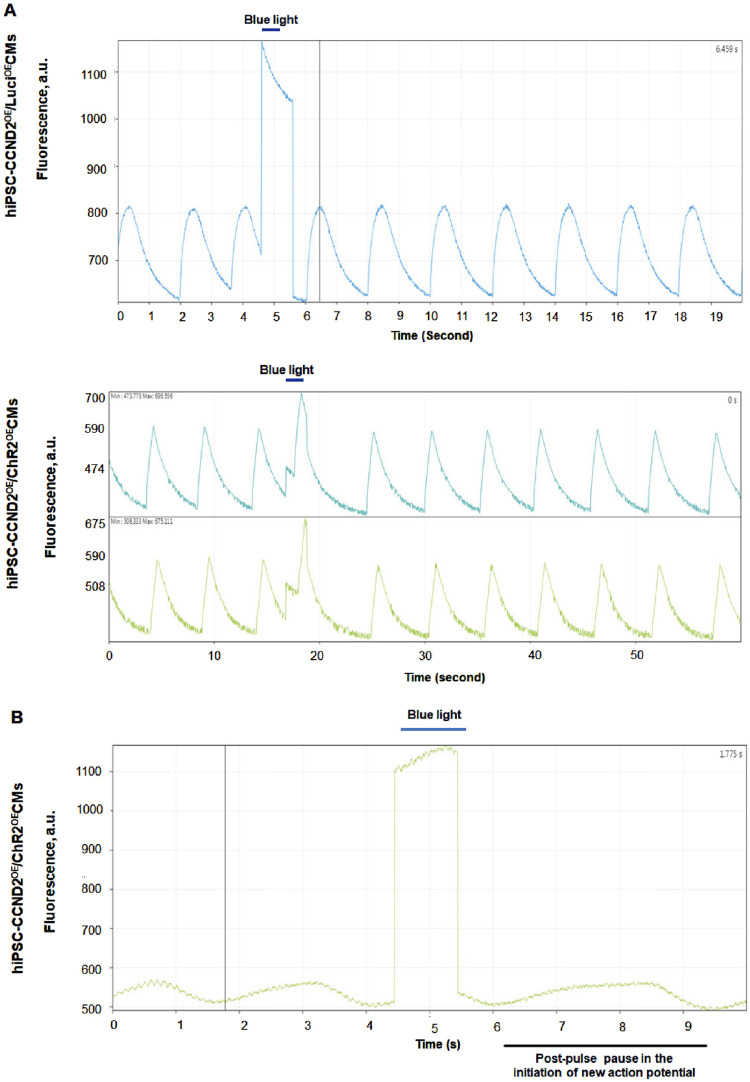
Functional effect of blue light (460 nm) on the human iPSC-CMs-ChR2. The light activation on control iPSC-CMs (not expressing ChR2) did not show any impact on the Ca^2+^ transient repolarization time **(A)**, top panel. The iPSC-CMs-ChR2 showed prolonged Ca^2+^ transient repolarization time **(A)**, lower panel and a pause in the initiation of new action potential **(B)** when activated with blue light (Joshi et al., 2022).

**FIGURE 5:**
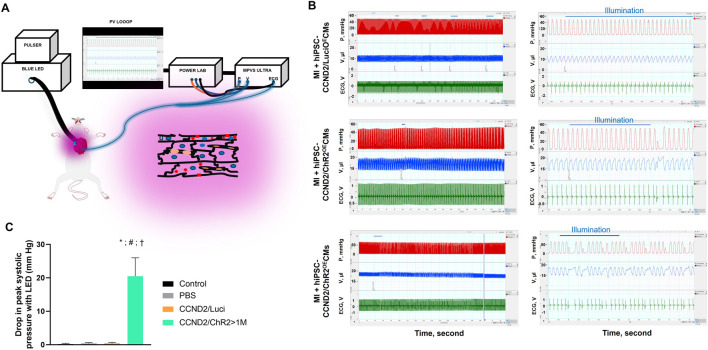
Functional changes in the myocardial functions using Optogenetics. A schematic illustration of the Optogenetic system utilized in the study **(A)**. Left ventricular pressure (red), volume (blue), and ECG (green) were simultaneously recorded, using P-V loop, from anesthetized, open-chest SCID mice 6 months after infarction and intracardiac injection of control (upper panel) or iPSC-CMs-ChR2 (middle and lower panels) before, during, and after blue light illumination. Left panels show tracings from the same animals at expanded time scales (LabChart Scales 500:1 and 20:1). The blue horizontal lines indicate the duration of light pulses **(B)**. At the onset of PVC, a significant reduction in systolic ventricular pressure (P_max_) was recorded with light activation in mice receiving ChR2 **(C)** (Joshi et al., 2022).

We recently reported optogenetic-based interrogation of action potential and calcium transients of iPSC-CM in a rapid, reversible and targeted manner (Joshi et al., 2022). Using CRISPR/Cas 9 genome editing tool, human iPSC-CMs were expressed with algal-derived transmembrane protein, ChR2 (iPSC-CMs-ChR2), that acts as light-gated cation channel (Joshi et al., 2022). Also, both control and iPSC-CMs-ChR2 were co-expressed with CCND2 and luciferin. The iPSC-CMs-ChR2 were- 1) labelled with calcium (Rhod-2 AM) or voltage-specific (RH237) dyes, 2) illuminated with blue light (460 nM, 1.6 mW/ mm^2^), 3) and their calcium transients and membrane voltage were optically mapped using MiCAM03 N256 Single Camera System (Scimedia Ltd., United States), and 4) data was analyzed using BV Workbench (Version 2.7.2, Scimedia, United States). We found that functional characteristics of calcium repolarization and excitability of action potential in iPSC-CMs can be successfully modulated non-invasively, reversibly and instantaneously by activating light-gated ChR2 ion channel expressed in the iPSC-CMs (Joshi et al., 2022). In brief, blue light activation on control iPSC-CMs (not expressing ChR2) did not show any impact on the Ca^2+^ transient repolarization time, but the iPSC-CMs-ChR2 showed prolonged Ca^2+^ transient repolarization time and a delay in the initiation of new action potential with blue light (Figure 4). In the same study, we also demonstrated that blue light was able to interrogate, in real-time, myocardial functions, such as pressure, volume and ECG, of the infarcted SCID mice receiving iPSC-CMs-ChR2, but not in the mice receiving the control iPSC-CMs (Figure 5), which underscores the translational impact of optogenetics in probing the electrophysiological characteristics at both cellular and organ-level (Joshi et al., 2022).

Apart from ChR2, other diverse group of transmembrane opsins, such as light-activated outwardly directed sodium pumps, yellow-light activated inwardly directed chloride pump (NpHR), calcium translocating channelrhodopsin variant (CaTCh) etc., have evolved over the past decade in cardiac research and medicine (Ferenczi et al., 2019; Joshi et al., 2019). In a recent study, Quach et al. demonstrated the scope of optogenetic-based dynamic current clamp platform to non-invasively tune the action potential of iPSC-CMs to a more mature phenotype (Quach et al., 2018). In brief, they introduced Archaerhodopsin TP009 (ArchT) opsin in iPSC-CMs to optically induce outward-current mimicking I_K1_, which is otherwise insufficient in the iPSC-CMs, and showed that 1) optically clamped iPSC-CMs showed more adult-like action potential morphology, similar to those when injected with I_K1_ by traditional patch clamp, and 2) the effects of I_Kr_ inhibition on iPSC-CMs were similar, when clamped optically or via means of standard electrodes, clearly depicting the scope of non-invasive and scalable optical dynamic clamping in drug screening and disease modeling (Quach et al., 2018). Notably, optogenetic tools have shown the ability not only in modulating the direction and flow of specific ion channel currents, but novel optogenetic tools can also successfully control intracellular signal pathways, such as Gs signaling, Wnt signaling and TGF-β signaling, with spatiotemporal precision (Joshi et al., 2019; Wu et al., 2023). Thus, optogenetics offers unique capabilities in probing functional and biological characteristics of iPSC-CMs; hence, it can exert an influential role in the forefront of arrhythmia research and therapeutic medicine.

## 6 The scope of iPSC-CMs beyond disease modeling

### 6.1 Personalized cardiotoxicity screening

The overall goal of this review paper is to examine the scope and challenges of using iPSC-CMs in arrhythmia modeling from basic science research and translational perspective. However, it should not be forgotten that human/patient-derived iPSC-CMs also hold significant potential on personalized cardiotoxicity screening and regenerative medicine. A clinical study on “Generation of Induced Pluripotent Stem Cell Derived Cardiomyocytes From Patients Exposed to Trastuzumab Therapy for Breast Cancer” (study location- Memorial Sloan Kettering Cancer Center, NY) uses skin biopsy of the patients, transform them into cardiomyocytes and aims to understand cardiotoxicity mechanisms in breast cancer patients, treated with anti-cancer drugs such as- Doxorubicin and Herceptin [NCT02772367]. Another study by Professor Joseph Wu and his team have also utilized human iPSC-CMs to understand the cellular mechanisms of trastuzumab-induced cardiac dysfunction (Kitani et al., 2019). They utilized 1) control iPSC-CMs which were derived from Stanford Cardiovascular Institute iPSC Biobank and 2) patient-specific iPSC-CMs which were derived from PBMCs of breast cancer patients, previously treated with trastuzumab and with or without drug-induced cardiac dysfunctions. They reported that clinically relevant doses of trastuzumab significantly impacted- 1) the contractile and Ca^2+^ handling characteristics and 2) mitochondrial function and cardiac energy metabolism of control iPSC-CMs. Interestingly, they demonstrated that iPSC-CMs generated from patients, who experienced severe cardiac dysfunctions with trastuzumab, were more vulnerable to trastuzumab treatment than the iPSC-CMs derived from patients, who did not exhibit cardiac dysfunction with trastuzumab therapy (Kitani et al., 2019). These studies underscore the translational impact of patient-specific iPSC-CMs in personalized drug-induced cardiotoxicity screening.

### 6.2 Cardiac regenerative medicine

Human/-patient-specific iPSC-CMs are also being utilized globally in multiple clinical studies to improve cardiac function and tissue regeneration. In clinical studies, autologous or allogeneic, iPSC-CMs are delivered either as single cells, spheroids, sheets, or patches to treat patients with heart failures or congenital heart defects [NCT05566600, NCT04696328, NCT04945018, NCT04982081, and NCT05647213]. A recent clinical trial by Professor Shigeru Miyagawa and his team (Osaka University, Japan) aimed to confirm the safety profile of allogenic iPSC-CMs transplantation in heart failure patients (Kawamura et al., 2023). In their study, they transplanted iPSC-CMs-based cardiac patches on the patients having 1) ischemic cardiomyopathy, 2) left ventricular ejection fraction of 35% or less and 3) heart failure symptoms of New York Heart Association class III or greater, despite existing therapies such as revascularization. Surgically, three iPSC-CM-based circular patches (each containing 3.3*10^7^ cells and diameter of 3.5 cm) were transplanted on the left ventricular anterior and the lateral wall and fixed. The patients were given immunosuppressants for 3 months after the surgery and were observed for up to a year. In the first 3 cases of the trial, they observed 1) improvement in heart failure symptoms, 2) no transplant-related adverse outcomes, such as tumor formation, during the observation period, and 3) 2 of the 3 patients showed improvements in left ventricular contractility and myocardial blood flow, demonstrating the feasibility and safety of utilizing allogenic human iPSC-CMs for treating heart diseases (Kawamura et al., 2023). Since the n-values of this study is low, it is hard to draw a definitive conclusion on the therapeutic efficacy and possible concerns of immune rejections on using such allogenic transplants. Nevertheless, novel developments on hypoimmunogenic and universal iPSC lines, using CRISPR/Cas9 (Zhao et al., 2020), can greatly circumvent concerns of immune rejection with allogenic iPSC-CMs and help to establish off-the-shelf iPSC-CMs-based allogenic patches, which can reduce the time and cost needed to generate autologous grafts and could lower dependency on the donor organs.

## 7 Summary and Future directions

Limited availability of human cardiac tissues necessitates the need of an accessible and scalable human-derived cell/tissue system that carries patient genetic information and expresses native cardiomyocyte-like structural and functional properties. Patient-derived iPSCs provide an inexhaustible source to obtain differentiated cells of all three germ layers in a reproducible manner. Specifically, current reprogramming and differentiation protocols have made it feasible to reliably obtain human iPSC-CMs that retain the genomic traits of the donor and exhibit the cellular and electrophysiological characteristics of the native cardiomyocytes. Thus, iPSC-CMs, derived from symptomatic or asymptomatic patients, are invaluable, accessible and scalable tools to understand the cellular and molecular mechanisms of channelopathies. Thus, in this review, we summarized several foundational and recent studies to provide in-depth knowledge on how patient-specific iPSC-CMs are utilized to model patient-specific arrhythmias that originated due to mutations on specific ion channel or dyad protein. We also highlighted recent advancements in CRISPR/Cas9 that have enabled us to establish patient-independent, variant-induced iPSC-CMs-based arrhythmia models. Next, we also examined developments on 3D models, such as iPSC-CMs-based cell sheets and patches, that capture *in vitro* electrophysiological characteristics in a more physiologically relevant manner, compared to 2D models. Notably, we also examined that such iPSC-CMs-based 3D patches have been transplanted in patients with ischemic cardiomyopathy to induce their myocardial tissue regeneration and improve myocardial functions. We further examined that a synergy of iPSC-CMs, gene editing, 3D models, all-optical, and high-throughput platform allow rapid acquisition of electrophysiological data in non-invasive and real-time manner, offering a platform to screen large number of emerging variants and investigate potential drugs/ therapies. Furthermore, we examined potential pitfalls of using iPSC-CM-based disease models that may arise due to differences in the expression level of ion channels or in their biophysical properties, highlighting the need to conduct iPSC-CM-based study and interpret the results with proper care. For example, iPSC-CMs are proarrhythmic when compared to their native counterparts; hence, such effects that arise due to the electrophysiological immaturity of iPSC-CMs should not be confused with the drug toxicity. This underscores the need to keep adequate experimental controls and isogenic controls of iPSC-CM for arrhythmia research. Importantly, we also examined that gene editing and optogenetics provide unique opportunity to tailor the functional characteristics of the ion channels or signaling components in the iPSC-CMs, allowing us to better understand the role of specific ion channel and signaling pathways in healthy or diseased conditions.

Our studies and genome-wide association studies have shown that arrhythmias arise not only due to mutations in ion channels but also due to mutations in genes encoding cytoskeletal proteins (β2 spectrin), adapter proteins (Ankyrin 2), ECM, fibrosis, cardio-genesis, and cell-cell coupling. Thus, it is crucial that *in vitro* arrhythmia modeling should address the complex disease phenotype thath can partly be achieved by differentiating iPSCs into different cardiac cells, culturing them in 3D (as organoids or patches) and providing them with appropriate biomechanical and electrical stimulus. Such biomimetic 3D model would allow to comprehend the specific role of cellular or signaling components in eliciting arrhythmias. In addition, integration of such 3D culture models with high-throughput screening platform would allow rapid characterization of the variants and their targets in a physiologically relevant manner. More importantly, such high-throughput-based *in vitro* iPSC-CM arrhythmia models, in conjunction with reliable gene editing technology and all-optical technology, would provide reliable disease templates to rapidly and reliably screen large number of emerging arrhythmogenic variants and test novel therapies, while simultaneously reducing the time and cost of pre-clinical research.

## Figures and Tables

**Table:** 

**iPSCs**	Induced pluripotent stem cells
**iPSC-CMs**	Induced pluripotent stem cell-derived cardiomyocytes
**SR**	Sarcoplasmic reticulum
**TT**	Transverse tubule
**E-C**	Excitation-contraction coupling
**CICR**	Calcium-induced calcium release
**NCX**	Sodium/calcium exchanger
**Na/K-ATPase**	Sodium/potassium ATPase
**InsP3R**	Inositol triphosphate receptor
**RyR2**	Ryanodine receptor 2
**BrS**	Brugada syndrome
**CPVT**	Catecholaminergic polymorphic ventricular tachycardia
**CASQ2**	Calsequestrin 2
**SQTS**	Short QT syndrome
**ICC**	Inherited cardiac channelopathies
**OSKM**	Oct3/4, Sox2, Klf4 and c-Myc
**LQTS**	Long-QT syndrome
**APD**	Action potential duration
**EADs**	Early afterdepolarizations
**DADs**	Delayed afterdepolarizations
**EHT**	Engineered heart tissues
**VUS**	Variants of uncertain significance
**CRISPRi/a**	CRISPR-based interference and activation system
**dCas9**	Deactivated Cas9
**ChR2**	Channelrhodopsin
**CTD**	Calcium transient duration
**EHM**	Engineered heart muscle
**T3**	Triiodothyronine
**Dex**	Dexamethasone
**NRVM**	Neonatal rat ventricular cardiomyocytes
**iPSC-CMs-ChR2**	iPSC-CMs expressing Channelrhodopsin
