# Supplementary material for: Human induced pluripotent stem cell-derived cardiomyocytes (iPSC-CMs) for modeling cardiac arrhythmias: strengths, challenges and potential solutions
Source: Front Physiol. 2024 Sep 12;15:1475152. doi: 10.3389/fphys.2024.1475152 (PMC11424716; doi:10.3389/fphys.2024.1475152)
Supplement: Supplementary file 1 [file Table1.pdf]

Supplementary Table 1. *A list of foundational and recent studies utilizing patient-derived/human iPSC-CMs to uncover the functional role of cardiac ion channels and dyad proteins in eliciting arrhythmogenesis.*

	Condition	Study	Gene Variant	Characteristics of mutant human iPSC-CMs compared to healthy controls
**Foundational studies on specific gene variant-induced iPSC-CM-based arrhythmia models**	BrS	(Kosmidis et al., 2016)	SCN5A p.(Arg1638X)	1. Significantly reduced V_{max}<br>2. Reduced peak I_{Na} density<br>3. Reduced cardiac AP upstroke velocity
	CPVT	(Fatima et al., 2011)	RYR2 p.(Phe2483Ile)	1. Increased DADs and arrhythmias when exposed to adrenergic agonists<br>2. Higher amplitudes and longer durations of spontaneous local Ca^{2+} release events
	LQTS	(Moretti et al., 2010)	KCNQ1 p.(Arg190Gln)	1. Prolonged APD<br>2. Reduction of I_{Ks}<br>3. Enhanced vulnerability to catecholamine-induced tachyarrhythmia, which is reduced with beta blockers
	SQTS	(El-Battrawy et al., 2018)	KCNH2 p.(Asn588Lys)	1. Shortened APD<br>2. Increased I_{Kr} and hERG
**Expansion and optimization of gene-variant induced iPSC-CM-based arrhythmia models**		(Selga et al., 2018)	SCN5A p.(Arg367His)	1. Reduction in I_{Na} peak current density<br>2. Accelerated recovery from Na channel inactivation<br>4. Similar changes I_{Na} properties observed across different iPSC-CM differentiation procedures
		(de la Roche et al., 2019)	SCN5A p.(Ala735Val)	1. Reduced cardiac AP upstroke velocity due to reduced I_{Na} inward<br>2. Right shift of Na activation curve, prolonged recovery from inactivation
	BrS	(Li et al., 2020)	SCN5A p.(Ser1812X)	1. Reduced peak I_{Na} density<br>2. Irregular Nav1.5 localization and decreased colocalization of Nav1.5 and Cx43<br>3. Reduced Nav1.5 expression<br>4. Reduced cardiac AP upstroke velocity

				5. Abnormal AP profile, including EADs and DADs<br><br>6. First demonstration of conduction slowing in BrS-CMs
		(Lu et al., 2023)	SCN5A p.(Arg620His)+ p.(Arg811His)	1. I_{Na} density reduced, lower NaV1.5<br><br>2. Beating interval variation
	CPVT	(Itzhaki et al., 2012)	RYR2 p.(Met4109Arg)	1. No observed difference in AP properties<br><br>2. Increased occurrence of DADs<br><br>3. Significant whole-cell Ca^{2+} transient abnormalities, which aggravated upon adrenergic stimulation but improved with β-blockers
		(Novak et al., 2012)	CASQ2 p.(Asp307His)	1. Prolonged APD<br><br>2. DADs and diastolic Ca^{2+} increase caused by β-adrenergic stimulation
		(Zhang et al., 2013)	RYR2 p.(Phe2483Ileu)	1. Caffeine-induced Ca^{2+} transients produced smaller I_{NCX}, showing smaller Ca^{2+} stores<br><br>2. Higher CICR gain<br><br>3. Increased diastolic Ca^{2+} level and spontaneous Ca^{2+} release
		(Acimovic et al., 2018)	RYR2 p.(Asp3638Ala)	1. Abnormal Ca^{2+} release under stress<br><br>2. Decreased Ca^{2+} transient amplitude under stress<br><br>3. Increase in systolic and diastolic aberrant Ca^{2+} events under stress<br><br>4. Leaky RYR2 channels under stress
	LQTS	(Itzhaki et al., 2011)	KCNH2 p.(Ala614Val)	1. Prolonged APD<br><br>2. Reduction of I_{kr} peak amplitudes at depolarization steps<br><br>3. Development of EADs
		(Egashira et al., 2012)	KCNQ1 1893delC	1. Prolonged Field potential duration (FPD)<br><br>2. Treatment of LQTS-iPSC-CMs with isoproterenol induces ventricular tachycardia-

				like arrhythmia, which was blocked by β-blocker
		(Ma et al., 2013, p. 3)	SCN5A p.(Val1763Met)	1. Increased late I_{Na} and APD prolongation<br>2. Shortened I_{Na} inactivation recovery
		(Malan et al., 2016)	SCN5A p.(Arg1644His)	1. Prolonged APD<br>2. High incidence of EADs<br>3. Accelerated recovery from Na current inactivation
		(Garg et al., 2018b)	KCNH2 p.(Thr983Ile)	1. I_{kr} density reduced<br>2. High diastolic Ca^{2+}
