# Supplementary material for: Human induced pluripotent stem cell-derived cardiomyocytes (iPSC-CMs) for modeling cardiac arrhythmias: strengths, challenges and potential solutions
Source: Front Physiol. 2024 Sep 12;15:1475152. doi: 10.3389/fphys.2024.1475152 (PMC11424716; doi:10.3389/fphys.2024.1475152)
Supplement: Supplementary file 2 [file Table2.pdf]

Supplementary Table 2. *Comparison of the electrophysiological, morphological, ultrastructural, and metabolic characteristics of iPSC-CMs versus adult cardiomyocytes.*

**Ion Channels, Currents**		**Genes that encode major pore-forming α-Subunit/s**	**Genes that encode minor β-Subunit/s)**	**Status of hiPSC-CM when compared to adult CM**		**References**
	Nav1.5<br>I_{Na} (I_{NaT}, I_{NaL})	SCN5A	SCN1B–SCN4B	Lower functional availability of Na channels		(Ma et al., 2011; Hoekstra et al., 2012; Garg et al., 2018a)
	Cav1.2<br>I_{CaL}	CACNA1C	CACNB2, CACNG	Robust expression of I_{CaL} and similar properties		(Ma et al., 2011; Hofmann et al., 2014; Garg et al., 2018a)
	Kv4.2, Kv4.3<br>$I_{to,f}$ (fast)	KCND2, KCND3	KCNE2-3, KCNIP1-2, DPP6	Slightly lower functional expression with a slow recovery of outward current from inactivation.		(Niwa and Nerbonne, 2010; Ma et al., 2011; Garg et al., 2018a)
	Kv7.1<br>I_{Ks} (slow)	KCNQ1	KCNE1	Similar current densities		(Lee et al., 2000; Moretti et al., 2010; Garg et al., 2018a)
	Kv11.1<br>I_{Kr} (rapid)	KCNH2 (hERG1)		Similar current densities		(Salama and London, 2007; Ma et al., 2011; Lahti et al., 2012; Garg et al., 2018a)
	Kir2.1 (Kir2.2/2.3)<br>I_{K1}	KCNJ2 (KCNJ12/KCNJ4)		Lower I_{K1} current density along with I_f contributes to spontaneous beating and relatively depolarized resting membrane potential.		(Zaritsky et al., 2001; Ma et al., 2011; Garg et al., 2018a)
**Electrophysiology**				**hiPSC-CMs**	**Adult CMs**	
	Conduction Velocity			10–20 cm/s	60 cm/s	(Denning et al., 2016; Herron, 2016)
	Gap Junctions			All over the cell membrane	Intercalated discs	(Wu et al., 2021)
	Resting Membrane Potential			−20 to −60 mV	−80 to -90 mV	(Denning et al., 2016; Karbassi et al., 2020)
	Ion Channel Activation	**Channel**	**Current**			
		$Na_v1.5$	I_{Na}	− 38.7 ± 0.3 mV<br>[mid activation voltage]	Atrial CMs: − 44.9 ± 0.5 mV<br>Ventricular CMs: − 43.4 ± 0.4 mV<br>[mid activation voltages]	(Goodrow et al., 2018)

		Ca_v1.2	I_{CaL}	-50 mV	Around -30 mV	
		Ca_v3.1, 3.2	I_{CaT}	-50 mV	~ -60 mV	(Grant, 2009; Uzun et al., 2016)
		Kir 2.1/2.2	I_{K1}	-50 mV	~ -40 mV	(Jeevaratnam et al., 2018)
		HERG	I_{Kr}	−21 ± 2 mV [V ½]	-40 to -30 mV	(Grant, 2009; Altrocchi et al., 2020)
	Ion Channel Inactivation					
		Nav1.5	I_{Na}	− 79.9 ± 0.9 mV	Atrial CM: − 82.5 ± 0.1 mV<br><br>Ventricle CM: − 74.4 ± 0.2 mV	(Goodrow et al., 2018)
		Cav1.2	$I_{Ca\text{-}L}$	-10 mV	~ -40 mV	(Grant, 2009; Uzun et al., 2016)
		Cav3.1, 3.2	$I_{Ca\text{-}T}$	-30 mV	Hyperpolarized	(Grant, 2009; Uzun et al., 2016)
		HERG	I_{Kr}	-40 mV [V ½]	+20 to +40 mV	(Altrocchi et al., 2020; Zequn and Jiangfang, 2021)
**Morphology**	Size			Smaller	Larger	(Yang et al., 2014; Ahmed et al., 2020)
	Shape			Circular	Rod-shaped	(Yang et al., 2014)
	Nuclei			Mononucleated (immature)	25% multinucleated	(Yang et al., 2014)
**Ultrastructure**	Mitochondria			Long, slender, and lacks mitochondrial cristae.	Ovular shaped, occupy 20-40% of the cell volume	(Wu et al., 2021)
	Sarcomere			1.85 ± 0.046 µm	1.91 ± 0.01 µm	(Lemcke et al., 2020)
	Z-line thickness			73.45 ± 1.24 nm	74.6 ± 1.85 nm	(Lemcke et al., 2020)
	T-tubule			Absent	Present	(Wu et al., 2021)
**Myofibrillar Isoform**	Titin			N2BA	N2B	(Yang et al., 2014; Wu et al., 2021)
	Myosin Heavy Chain			β≈α	β>>α	(Yang et al., 2014; Wu et al., 2021)

**Metabolism**				Mainly glycolysis for energy production in immature iPSC-CMs	Fatty acid/β-oxidation	(Vučković et al., 2022)
